# Catch basin larvicide treatments impact adult mosquito West Nile virus vector species in metropolitan Milwaukee, WI, U.S.A.

**DOI:** 10.1371/journal.pone.0342150

**Published:** 2026-04-15

**Authors:** Haley E. Johnson, Justin E. Harbison, Jessica L. Hite, Bradley J. Tucker, J. Mitchell Kirsch, Susan Paskewitz, Lyric C. Bartholomay

**Affiliations:** 1 Department of Pathobiological Sciences, University of Wisconsin-Madison, Madison, Wisconsin, United States of America; 2 Loyola University Chicago, Maywood, Illinois, United States of America; 3 Department of Entomology, University of Wisconsin-Madison, Madison, Wisconsin, United States of America; University of Minnesota, UNITED STATES OF AMERICA

## Abstract

Roadside stormwater catch basins are frequently treated with larvicides in metropolitan areas because they are ideal larval growth sites for West Nile virus (WNV) vector species *Culex pipiens* Linnaeus and *Cx. restuans (Theobald)*. Despite the wide-spread implementation of catch basin treatments in integrated vector management programs, there is little entomological evidence to suggest catch basin larvicide treatments reduce adult mosquito abundance and thereby impact WNV risk. We evaluated the impact of treating catch basins at three of four sites (700–1000 basins each) with a formulation of *Lysinibacillus sphaericus* using a stepped-wedge cluster trial approach in metropolitan areas in Wisconsin, U.S.A in 2019. Treatment effects were measured by evaluating immature stage mosquito abundance in catch basins and general additive models (GAM) to analyze changes of the integrated adult female *Culex* spp. abundance and population stability sampled from host-seeking and gravid mosquito traps. We observed catch basin treatments to effectively suppress immature stages in catch basins (94% reduction of pupae). The GAM found catch basin treatment duration to significantly reduce the integrated abundance of gravid *Culex* spp. mosquitoes, with a total mean percent reduction of 37% at treated sites. Treatment duration was also observed to impact the stability of the gravid *Culex* spp. integrated abundance, with differences between untreated and treated sites, and among sites that differed in treatment duration. These results support the treatment of catch basins with biorational larvicides as a mosquito management tool for WNV vector species in similar metropolitan habitats provided adequate suppression of immature stage mosquitoes.

## Introduction

West Nile virus (WNV) is a mosquito-borne virus that impacts human and veterinary health world-wide [1]. WNV is naturally maintained in an enzootic cycle between avian and mosquito hosts but can spillover to humans, resulting in significant morbidity and mortality [2]. Mosquitoes in the genus *Culex* (Diptera: Culicidae) are the primary vectors for WNV; germane to this study, *Cx. pipiens* Linnaeus and *Cx. restuans* (Theobald) are two principal species for enzootic and endemic transmission in the Midwest and Northeast United States [3,4]. Vector control of *Culex* vector spp. is the principal strategy to prevent human WNV transmission, with a large majority of efforts dedicated to larval control [5]. Larval control is a proactive strategy to prevent adult mosquito populations and virus amplification from achieving levels that could lead to disease outbreaks [6]. Intervention at this life stage is key to integrated mosquito management (IMM), particularly because there is evidence of adulticide insecticide resistance in *Culex pipiens* in this area, and adult mosquito control yields variable impacts on adult abundance [7,8]. In addition, larvicides often are more appealing than adulticides because many larvicide formulations are biorational, have greater target-specificity, pose less of a risk to non-target organisms, and relatively easy to apply because most do not require special application equipment [9].

Many larvicide products are formulated and packaged specifically for use in stormwater catch basins, infrastructures that intercept water from roads and other drainage courses. Catch basins are ubiquitous in metropolitan areas and often includes a settling sump to prevent debris, landscape refuse, and sediments in the runoff from entering the water management system [10]. Because stormwater catch basins hold water and organic material for extended periods, these structures provide optimum cues for ovipositional behavior of filth-breeding mosquitoes, and suitable conditions for larval growth for *Cx. pipiens* and *Cx. restuans* [11,12]. Therefore, catch basin larvicide applications are a priority for many mosquito abatement districts for WNV prevention and control [13,14]. Indeed, catch basins have long been targets of larval control efforts in metropolitan settings [15–17].

Although the effectiveness of various larvicide formulations to suppress *Culex* spp. larval abundance in catch basins is well documented, our understanding of whether larval suppression substantially reduces adult mosquito populations (and thereby reduces WNV risk) remains fragmentary [12,18,19]. Quantifying the impact of larvicide applications on subsequent adult mosquito populations in the wild remains challenging. Mosquito populations fluctuate seasonally and are sensitive to climatic conditions resulting in significant variability in mosquito abundance during the course of a study. Such seasonal effects can make it challenging to discern treatment effect from natural population fluctuations, especially without an untreated site for comparison [20]. In many communities with established mosquito abatement capacity, it is not possible to incorporate an untreated control group into studies of intervention effectiveness [21]. It is also challenging to conduct intervention studies on a scale similar to that used by mosquito abatement programs.

As a critical first step in addressing the above concerns, we designed a study to assess catch basin larvicide applications impact on adult *Cx. pipiens* and *Cx. restuans* abundance in the metropolitan area of Milwaukee, WI, U.S.A. using a stepped-wedge cluster trial (SWCT) approach. The SWCT is a variation of a crossover study design where each cluster receives treatment at different time points in a stepped-wedge fashion. Each cluster had before and during treatment observations, while one cluster remains untreated the entire duration [22,23]. A SWCT approach was selected because of its robustness in comparison to observational studies, incorporating multiple points for treated and untreated comparison within and among clusters. This approach was also selected for its practicality, gradually rolling out the treatment among sites [20]. The Milwaukee area provided an ideal study setting because it does not have established mosquito abatement districts and has only been subjected to sporadic catch basin larval control efforts. Therefore, we could incorporate both treated and untreated controls in our study design. Additionally, sites chosen in the Milwaukee area had comparable catch basin density per square mile to nearby metropolitan areas in the surrounding Chicago, IL area, meaning this study was conducted at a scale that is relevant to mosquito abatement programs that conduct annual community-wide catch basin treatments [19].

## Materials and methods

### Study location

**Fig 1:**
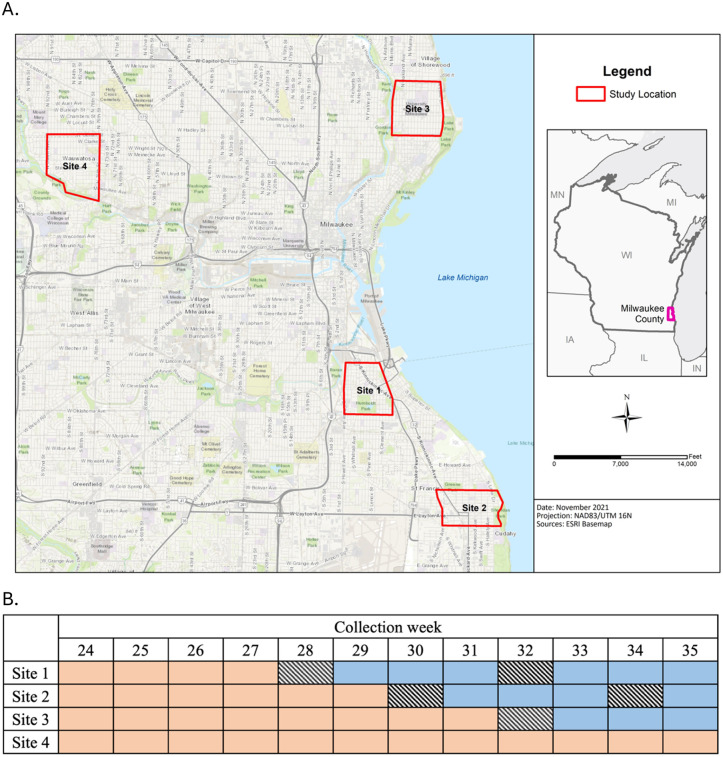
(A) Locations of four 2.59-km^2^ study sites within Milwaukee County, WI, U.S.A. **(B) Stepped-wedge cluster study design**. Orange periods represent weeks catch basins were not treated. Diagonal boxes represent weeks catch basins received applications of *L. sphaericus (*VectoLex® FG*)*. Blue periods indicate weeks catch basins were in treated conditions.

This study took place in Milwaukee County in southeastern Wisconsin, U.S.A., June through September 2019. Four 2.59 km^2^ study sites were selected based on landscape composition similarity and comparable numbers of catch basins (Fig 1A). Additionally, we conducted a pilot study the year prior in 2018 at two of the four selected sites and confirmed that catch basins in these areas are highly productive for mosquito larvae (S1 Fig). Site 1 is located in the Bay View neighborhood, south of downtown Milwaukee and has 974 catch basins. Site 2 is in the city of Cudahy, located south of Bay View with 748 basins. Site 3 is at the University of Wisconsin-Milwaukee campus, located in a residential neighborhood on the upper east side of Milwaukee with 806 basins. Site 4 is in the city of Wauwatosa, a suburban neighborhood that is more geographically distant from the other three sites and has 782 basins. To the best of our knowledge, the catch basins in all of these study sites are separate from the municipal waste water systems. To avoid sampling mosquitoes migrating from other sites, all sites were located a minimum of 8 km apart, which is substantially greater than the observed mean *Cx. pipiens* flight distance from its catch basin breeding habitat [24].

### Catch basin larvicide applications and evaluations

To evaluate larval and pupal abundance in catch basins in all four sites, mosquito larvae and pupae from 30 catch basins each week at each site were sampled, on the same day, from weeks 24–35 (9 June to 31 August) (S2 Fig). Catch basins were randomly selected for sampling based on safety and ease of access, and no basin was evaluated for consecutive weeks. Two dips were made at each catch basin using a standard 350 mL dipper and the presence or absence of early-instar (first or second instar) larvae, late-instar (third or fourth instar) larvae, and pupae was recorded according to an established ‘pass-fail’ protocol used to test the effectiveness of catch basin larvicide treatments [18]. Any pupae observed were also counted and recorded as a metric for catch basin productivity. To understand what species were present in basins, any live late-instar larvae were collected, transferred to a 15 ml conical tube with ethanol, labeled with date, catch basin number, and site. Larvae were later identified to species based on morphological characteristics [25].

Catch basins were treated with 20 g of VectoLex® FG (Valent BioSciences LLC, Libertyville, IL, active ingredient, 7.5% *Lysinibacillus sphaericus* strain ABTS-1743), a granular formulation that is labeled up to 28 days of control. *L. sphaericus* (formerly known as *Bacillus sphaericus*) is a microbial control agent found naturally in soils, is highly target-specific, and effective at suppressing mosquito larvae in polluted aquatic environments rich with organic material, like catch basins [9]. Application rates used in this study were selected based on the label rate and to be comparable to those used by mosquito abatement programs elsewhere and were vetted and proven to effectively suppress larvae in a 2018 pilot study (S1 Fig) [10,18]. If a catch basin was noted as not containing any water, it was not treated or sampled during the course of this study.

Catch basin larvicide treatments were made following a stepped-wedge cluster trial (SWCT) design. Sites 1, 2, and 3 received larvicide applications successively two weeks apart, while Site 4 remained untreated (Fig 1B). Subsequent larvicide applications were made every 30 days after the initial application to ensure larval abundance in catch basins continued to be suppressed the remaining study duration. Maps detailing the location of catch basins were provided by the respective public works departments to ensure we visited all accessible basins for complete treatment coverage of catch basins. The process of applying larvicide to catch basins to each site took place on one day.

A total of approximately 104 kg of VectoLex® FG was used to treat 5,054 catch basins among three sites during six application events. Site 1 received three treatments at weeks 28 (9 July), 32 (8 August), and 33 (16 August) for an estimated treatment coverage of 50 days. Site 2 received two larvicide applications at weeks 30 (25 July) and 34 (22 August) for an estimated treatment coverage of 35 days, and Site 3 received one larvicide application at week 32 (6 August) for an estimated treatment coverage of 22 days (Fig 1B). Site 1 received an additional larvicide application at week 33 because inspections after the week 32 application revealed that 53.33% (16 of 30 basins) of catch basins had late-instar and or pupae (S4 Table), surpassing the 25% threshold set by Nasci et al. [18]. Site 4 was untreated for a duration of 89 days. All larvicide applications were conducted under the approval of the State of Wisconsin Department of Natural Resources Pest Control Pollutant Discharge Elimination System Mosquito & other Flying Insects Permit No. WI-0064581.

### Adult mosquito surveillance

To sample the gravid and host-seeking adult *Culex* spp. abundance, two different traps were used. The gravid trap uses a baited attractant to collect gravid *Culex* spp., which are mosquitoes near the end of the gonotrophic cycle and ready to oviposit [1]. Conversely, CDC-baited light traps are used to collect host-seeking mosquitoes byway of emitted carbon dioxide (CO_2_) and are less specific to *Culex* spp. than gravid traps [1]. Each site had 10 CDC gravid traps (Model 1712, John W. Hock Co., Gainesville, FL) baited with an alfalfa pellet infusion to serve as a suitable oviposition site and attract gravid mosquitoes. Each site also had five miniature CDC-baited light traps (Model 512, John W. Hock Co., Gainesville) that were placed in proximity to 5 of the 10 gravid traps in the site (S2 Fig). Each CDC-light trap was baited with CO_2_ released at 0.5 kg/ day from a 9.07 kg tank using a pressure regulator (Biogents, Regensburg, Germany). Trap placement was selected based on landowner cooperation and was aggregated as near to the center of the site as possible to avoid potential catch bias from outside mosquito populations, according to the average flight distance (1.15 km) of *Cx. pipiens* from its catch basin breeding habitat [24]. Consent from each landowner was provided beforehand for trap placement and mosquito collection.

In total at sites 1, 2 and 4, 27 traps were placed in residential yards, two traps at local businesses, one trap at a community garden; at Site 3, all 15 traps were dispersed throughout the University of Wisconsin- Milwaukee campus (S2 Fig). Traps were operated for four consecutive trap nights weekly. Trap contents were collected every 24 h from weeks 24–35. Failed trap events were excluded from analysis*.*

All adult female mosquitoes were counted and identified to species with dichotomous keys [26]. *Culex pipiens* and *Cx. restuans* collected in CDC-baited light traps were morphologically separated using the characteristics outlined by Ferreira-de-Freitas et al. (2020) [27]. If the specimen was *Cx. pipiens* or Cx. *restuans*, but was too damaged to differentiate, it was recorded as *Cx. pipiens/restuans*. Gravid *Cx. pipiens* and *Cx. restuans* were not separated and were recorded as *Cx. pipiens/restuans* because over 157,000 gravid *Culex* spp. were collected, and therefore *Culex* spp. separation was not feasible.

### Statistical analysis

All statistical analysis was performed in R version 4.1.2 [28]. We tested the hypothesis that the average number of adult *Cx. pipiens* and *Cx. restuans* significantly decreased as the treatment duration (and therefore the number of larvicide applications) increased. To determine whether treatments differed across the different life history stages of adult *Culex* spp*.*, abundance data from gravid and CDC-baited light traps were evaluated separately. Not all individuals captured in the CDC-baited light traps were able to be speciated as either *Cx. pipiens* or *Cx. restuans*, therefore, to avoid excluding a subset of mosquitoes in the analysis, undifferentiated *Cx. pipiens/restuans* and differentiated *Cx. pipiens* and *Cx. restuans* were combined as *Cx. pipiens/restuans*. We used generalized additive models (GAM) with the integrated (area under the curve) abundance and stability (the standard deviation of abundance) of different mosquito species over the course of the sampling period as the response variable. Treatment duration was used as the main effect and date of first treatment and site as covariates with the number of functional traps as an offset. We also explored more complicated random effects structure (e.g., random slope and intercepts and time dependence), but our limited sample size precluded these more complex models. For all GAM models we considered negative binomial and Poisson distributions to account for heteroscedasticity. We used Akaike information criteria (AIC) for model selection. We then examined the selected models using the residual diagnostics, summary, predict. and assessed significance of the fixed effects using Wald *x*^2^ statistics (packagse: Car, mgcv).

For *post hoc* analyses between sites, we used least square means with planned contrasts and Bonferroni correction for multiple comparisons. The percent change in the adult abundance of each species and trap type was quantified by subtracting the total abundance at the treated site from the untreated site and then dividing by the total abundance at the treated site and multiplied by 100. Adult abundance data from weeks 24–26 were excluded from analysis because untreated catch basins at this time had minimal immature stage mosquitoes present. Adult abundance data collected one to seven days post initial treatment at each site was also excluded from analysis, as this time period was considered neither treated nor untreated because of the natural delay associated with larvicide treatments. Pearson’s correlation was used to compare the portion of *Cx. pipiens*, and subsequently *Cx. restuans* larvae collected from untreated catch basins to the proportion of each species that was able to be differentiated collected in the CDC-baited light traps.

## Results

### Catch basin evaluations and larval abundance

**Fig 2:**
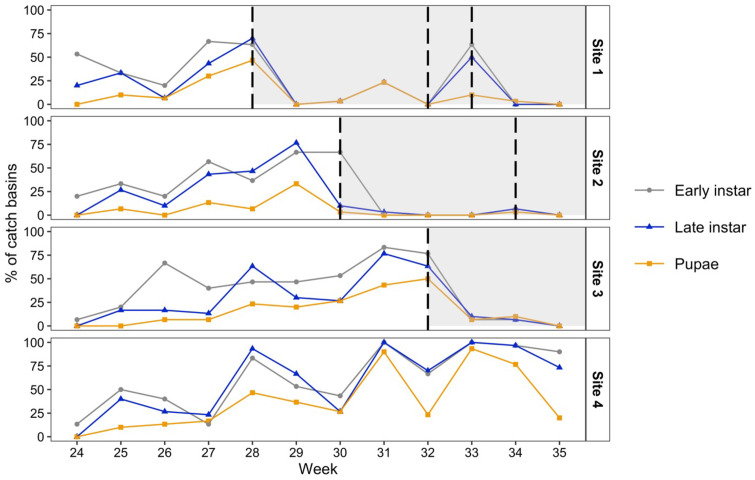
Weekly percent of catch basins inspected with early instar mosquito larvae (first and second), late instar larvae (third and fourth), and pupae at each site. Timing of catch basin larvicide applications with *L. sphaericus* illustrated by dashed vertical black lines. Grey frames show when site was under treated conditions.

Catch basin larvicide applications were conducted in neighborhoods using a SWCT approach for 4 sites as follows: Site 1 (Bay View) had sufficient larvicide treatment to suppress larval abundance in catch basins for approximately 50 days, Site 2 (Cudahy) for 37 days, Site 3 (UW-Milwaukee campus) for 25 days, and Site 4 (Wauwatosa) was left untreated for 89 days (Fig 2). The presence of early instar, late instar, and pupae varied by week, site, and treatment status. In the absence of any catch basin treatments, Site 4 continued to have larvae and or pupae present the duration of the study. In total, 2,942 late-instar larvae were collected from untreated catch basins, of which 70.3% (n = 2,067) were *Cx. restuans* and 29.5% (n = 869) *Cx. pipiens* (S3 Table).

Weekly pass/fail catch basin evaluations conducted after larvicide treatments of *L. sphaericus* (VectoLex® FG) showed that the mean number of pupae in treated catch basins decreased by 94.6% compared to untreated catch basins. The mean number of pupae per untreated catch basin was 9.20 ± 0.82 (mean ± SEM) (n = 1936 pupae in 210 basins) during weeks 29–35. The mean pupae per treated catch basin was 0.59 ± 0.42 (n = 266 pupae in 450 basins). Of the 266 total pupae observed in treated catch basins, 178 pupae were observed from three catch basins located in the same site and week. In total the number of catch basins observed with late-instar larvae and or pupae present was 8.0% (36 of 450 basins) in treated catch basins, compared to 77.14% (162 of 210 basins) in untreated catch basins. A summary of immature stage mosquitoes observed during weekly catch basin evaluations at each site are provided in the Supplemental Information (S4 Table).

### Adult mosquito abundance

**Fig 3:**
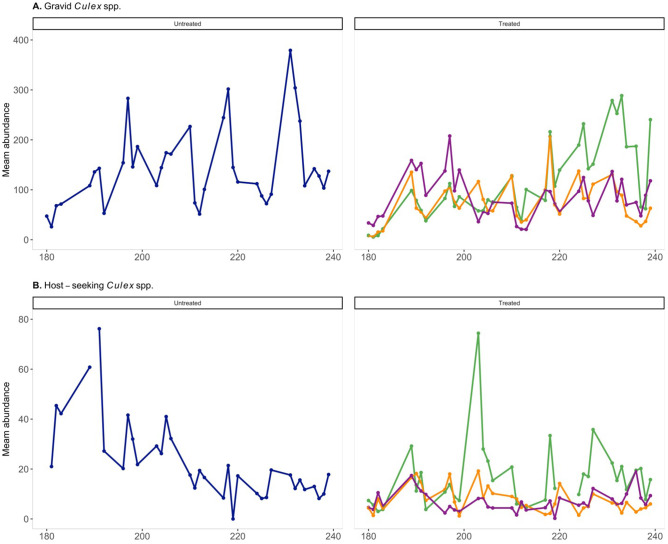
Mean daily (A) gravid *Cx. pipiens* and *Cx. restuans* abundance, (B) female host-seeking *Cx. pipiens* and *Cx. restuans* abundance captured in baited CDC-light traps. Each site differed in length of catch basin larvicide treatments ranging from not treated to fifty continuous days.

There was a considerable amount of variability within and among sites, as some trap locations consistently yielded more mosquitoes than others (Fig 3). Across all four sites, 244,278 *Culex* spp. mosquitoes were captured in gravid traps, and 15,806 *Culex* spp. mosquitoes were captured in CDC-baited light traps. Gravid traps collected more *Culex* spp. than CDC-baited light traps, 98.28% (244,278/248,549) and 50.34% (15,806/31,401) of trap contents were *Culex* spp. respectively in each trap type. A weekly summary of adult mosquito abundance data for each trap type is provided in the Supplemental Information (S5 Table).

In total, 77.9% (12,325/15,806) of host-seeking (mosquitoes collected in the CDC-baited light traps) *Culex* spp. mosquitoes were differentiated as either *Cx. pipiens* or *Cx. restuans*. Of these, there were approximately seven-fold more host-seeking *Cx. restuans* (10,801) collected and identified, as compared to *Cx. pipiens* (1,524). The proportion of each species collected and able to identified as adults in CDC-baited light traps significantly correlated to the proportion of each species collected as larvae in untreated catch basins (Site 1: r = 0.941, P = 0.002, n = 5 weeks; Site 2: r = 0.873, P = 0.005, n = 7 weeks; Site 3: r = 0.908, P = < 0.001, n = 9 weeks; Site 4: r = 0.926, P < 0.0001, n = 12 weeks).

### Adult abundance post- catch basin treatments

**Fig 4:**
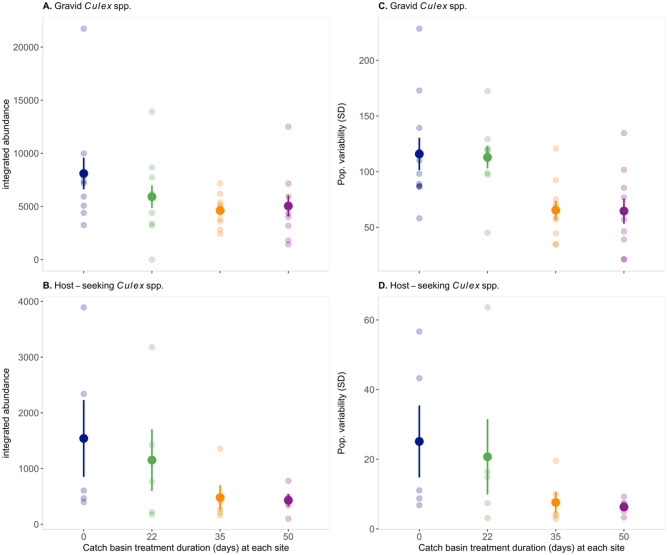
Integrated abundance of (A) gravid *Cx. pipiens* and *Cx. restuans*, (B) female host-seeking *Cx. pipiens* and *Cx. restuans.* **Population variability (standard deviation) of the abundance of (C) gravid *Cx. pipiens* and *Cx. restuans* and (D) female host-seeking *Cx. pipiens* and *Cx. restuans*.** Each site differed in length of catch basin larvicide treatments ranging from not treated to fifty days.

Overall, analyses from the GAM model suggest that treatment duration (total days catch basins were treated with *L. sphaericus* at each site) significantly reduced the integrated abundance (area under the curve) of gravid *Culex* spp. (not differentiated *Cx. pipiens* and *Cx. restuans*) (*x*^2^ = 4.258, P = 0.0391) (Fig 4A) (S6 Table). The gravid abundance at all treated sites (Sites 1, 2, and 3) was found to have a mean percent change of 37.85% (SD ± 13.96) compared to the untreated site (Site 4). No significant differences of the integrated abundance of host-seeking *Culex* spp. (*x*^2^ = 0.117, P = 0.732) were found (Fig 4B) (S6 Table). In the post-hoc analysis, we were unable to detect significant differences of the integrated density between sites for both gravid and host-seeking *Culex* spp. abundance (data not shown, all P-values > 0.05).

Additionally, catch basin treatment duration significantly altered the stability (standard deviation of the abundance) of gravid *Culex* spp. (*x*^2^ = 5.26, P = 0.0218) (Fig 4C) (S7 Table). The population stability of gravid *Culex* spp. at the 35-day treated site was significantly different than the untreated site (P-value = 0.021), and the 22-day treated site (P-value = 0.040). The population stability of the gravid abundance at the 50-day treated site was significantly different than the untreated site (P-value = 0.018), the 22-day treated site (P-value = 0.035), but not the 35-day treated site (P-value > 0.05). Treatment duration was not found to significantly affect the population stability of host-seeking *Culex* spp. (GAM: *x*^2^ = 0.532, P = 0.466) (Fig 4D) (S7 Table).

## Discussion

Catch basin larvicide applications are a widely practiced vector control strategy to reduce larval mosquitoes and, therefore, the abundance of adult mosquitoes. However, it is unclear if the effort and resources expended for catch basin control indeed equate to impacts on WNV infection in adult mosquitoes or incidence of WNV in people. Further, ecological theory and empirical studies demonstrate that numerous ecological mechanisms like density-dependence, fecundity overcompensation, or stage-structured competition can produce situations where reducing one stage (e.g., mosquito larvae) could result in higher densities of another stage (e.g., adult mosquitoes) [29]. Thus, treatment could be ineffective or even backfire, leading to unintended increases in adult mosquitoes. Understanding these cross-scale connections therefore carries important implications for both basic biology, public health, and economics, but there are few studies that have explored the impacts of larvicides on adult populations.

In Atlanta, GA, no treatment impact was observed on adult *Culex* spp. abundance or WNV infection prevalence after > 90% larval/pupal suppression was achieved in catch basins (56 and 134 catch basins at two sites) treated for approximately four weeks in urban parks [30]. McMillan et al. [31] evaluated the impact of community-wide applications of *L. sphaericus* (VectoLex® FG) in two coastal Connecticut towns (each with ~ 5000 and 8000 basins, one with a single 4-week treatment, the other with discontinuous treatment over 10 weeks); no treatment impact was detected on adult *Culex* spp. abundance or WNV infection prevalence. In this study, we evaluated the effectiveness of catch basin treatments to reduce larval and adult *Cx. pipiens* and *Cx. restuans* abundance in metropolitan Milwaukee, WI, U.S.A., where a community-wide study was possible in the absence of existing vector control efforts. Four study sites were selected where we could implement a stepped-wedge cluster design for catch basin treatments, such that one site was treated for 22 days, one for 35 days, one for 50 days, and one site remained without treatment for 89 days.

### Impacts of catch basin treatments on immature-stage abundance

In our study sites and based on our treatment approach, we observed extensive reduction of larval and pupal abundance in catch basins after *L. sphaericus* (VectoLex® FG) applications (Fig 2**).** We achieved approximately 94.6% reduction of pupal abundance in catch basins overall at treated sites, note that three samples skewed this result. Pupae are the last immature stage before adult emergence, therefore are a better indicator for adult emergence vs presence of early or late-instar larvae [32]. Therefore VectoLex® FG applications were highly effective, and our data corroborate previous reports that catch basin larvicide applications effectively reduce *Culex* spp*.* immature-instar abundance (e.g., [18,33]). Because we evaluated catch basins routinely before and after treatments and observed significant larval and pupal reduction (Fig 2), we are confident that treated catch basins were not a productive source for mosquito emergence in treated sites in this study.

### Impacts of catch basin treatments on the adult abundance

The overall integrated abundance of gravid *Culex* spp*.* significantly decreased as the treatment duration, and therefore the number of larvicide applications increased (Fig 4A). Our results support the conclusion of previously developed stage-structured mechanistic models, that continuous application of larvicides have a greater impact than short-term larval control strategies [34]. Gravid traps are the preferred trap type for West Nile virus surveillance because in this physiological state, gravid (egg-bearing) mosquitoes have had at least one potentially infectious blood-meal [13] as anautogenous *Cx. pipiens and Cx. restuans* require a blood-meal to become gravid [35]. *Culex pipiens* and *Cx. restuans* are primarily ornithophilic but do occasionally feed on humans [3,36]. Therefore, it is assumed that a decline in the integrated abundance of gravid *Culex* spp. mosquitoes reduce the entomological risk [37].

We were unable to detect significant differences in the integrated abundance of *Cx. pipiens* and *Cx. restuans* when examined separately from the CDC-baited traps. The contrast between the results from the gravid and CDC-baited traps likely arises due to the pronounced variation across trap types. As expected, substantially fewer *Culex* spp. mosquitoes were collected in the CDC-baited light traps than gravid traps (Fig 3). On average, gravid traps captured 697.81 *Culex* spp. mosquitoes per trap event compared to the 82.8 captured per trap night for CDC-baited light traps. Given this stark contrast in trap counts between trap types, it is likely that too few host-seeking mosquitoes were captured to detect a difference.

Treatment duration significantly altered the stability (standard deviation of abundance) of the gravid *Culex* spp. abundance. More specifically, longer treatment durations resulted in increased stability (lower variation) relative to the shorter 22-day treatment and non-treated site (Fig 4). Increased stability can help protect populations from the risk of going extinct through stochasticity, small population sizes, and Allee effects. Increasing population stability is, therefore, a central goal of conservation management focused on endangered species. Could reducing population stability present an alternative or additional goal of vector management? While answering this question and examining how treatment duration influences the long-term viability of mosquito populations is beyond the scope of this current study, it does present an interesting avenue for future empirical studies conducted over long-time frames.

The complexity of mosquito population dynamics is driven by a variety of environmental and climatic factors [38]. This inherent complexity makes it difficult to evaluate the effectiveness of vector control strategies. To make things even more complicated, in Wisconsin, as well as in other locations, there are two principle WNV vector species with distinctive bionomic traits. *Culex restuans* are more abundant in the spring and early summer, while *Cx. pipiens* populations peak late-summer [39,40]. Although *Cx. pipiens* and *Cx. restuans* are both abundant in metropolitan areas, *Cx. restuans* are more frequently found in forested areas as compared to *Cx. pipiens* [41,42]. However, because adults of these species are morphologically difficult to discern, *Cx. pipiens* and *Cx. restuans* are grouped for arbovirus surveillance, muddling the distinct vector ecology of these species [27,37]. Given this information, it’s difficult to discern a treatment impact from natural population variability and could be especially challenging in areas that have both *Cx. pipiens* and *Cx. restuans* present.

Despite this inherent noise in the data, it is possible that we were able to detect an impact on the integrated abundance of adult gravid *Culex spp.* from the number of larvicide applications each site received because of the benefit of the chosen study location. In this study, we were able to conduct community-wide catch basin treatments for *Cx. pipiens* and *Cx. restuans* control in sites where no larval control was taking place. It can be difficult to justify a reduction of treatments at places that routinely receive catch basin treatments [21]. That said, future studies in locations that routinely treat basins could use a different iteration of the stepped-wedge design where one site does not remain without treatment, and instead receives treatment last, which would still provide within and among site comparison of different treatment durations [20]. Additionally, all four study sites had a similar relatively high density of basins per km^2^. Subsequent impacts on the adult vector species abundance from catch basin larvicide applications is likely dependent on the density of basins. In this study, treated sites had approximately 251 to 326.8 catch basins per km^2^. Catch basins may be less important to local adult mosquito production at locations with lower densities. For instance, no treatment impact was observed on the adult mosquito abundance at locations with 123.6 catch basins or less per km^2^ [31]. Further investigation is needed to determine the optimum density of catch basins that need to be treated for maximum impact on the adult vector species abundance.

Using a SWCT approach, we evaluated the impacts of *L. sphaericus* (VectoLex® FG) catch basin treatments across three of four sites to maximize the number of treated and untreated comparisons. This approach enabled a robust assessment of treatment effects on immature and adult mosquito abundance under operational conditions. We observed extensive reductions in immature stage abundance in catch basins after treatment and an impact on the gravid integrated abundance as well as population stability related to treatment duration. Notably, in this study, a 94.6% reduction of pupae in catch basins was associated with a 37% reduction in the adult gravid *Culex* spp. integrated abundance. These findings indicate a measurable downstream effect on adult gravid mosquito abundance, a critical entomological indicator for assessing human risk of WNV. Given that adult surveillance metrics are often the most feasible indicators for resource-limited settings, this study underscores the value of larval control interventions in reducing potential WNV transmission. The operational implications of these results are significant for guiding targeting larval management strategies in urban areas for two mosquito species of epidemiological importance.

## Supporting information

S1 Fig Catch basin weekly inspections in 2018. Weekly percent of catch basins inspected with early instar (first and second instar), late instar (third and fourth instar), and pupae in 2018. Catch basin larvicide applications with *L. sphaericus* illustrated by dashed vertical black lines and grey frames display when site was under treated conditions.
(PDF)

S2 Fig Adult mosquito trapping locations and treated catch basins in 2019. Adult mosquito gravid (blue triangle) and baited-CDC light trap (blue square) trapping locations. Inspected subset of catch basins (red circle) used in weekly evaluations; catch basins not visited in weekly evaluations not displayed in figure. (A) Site 1, Bay View, WI. A total of 952 catch basins were identified and treated within the 2.59 – km^2^ site boundary. (B) Site 2, Cudahy, WI. A total of 748 catch basins were identified and treated within the 2.59 – km^2^ site boundary. (C) Site 3, University of Wisconsin-Milwaukee campus, Milwaukee, WI. A total of 806 catch basins were identified and treated within the 2.59 – km^2^ site boundary. (D) Site 4, Wauwatosa, WI. A total of 782 catch basins were identified and treated within the 2.59 – km^2^ site boundary.
(PDF)

S3 Table Summary of late-instar mosquito larvae collected weekly from untreated catch basins located within the four 2.59 – km^2^ study sites in the greater Milwaukee, WI area in 2019. (DOCX)

S4 Table Weekly catch basin inspections in 2019. Number of observed catch basins with early-instar larvae (first and second), late-instar larvae (third and fourth), pupae, and number of pupae observed during weekly inspections at each of the four 2.59 – km^2^ sites located in the greater Milwaukee, WI area in 2019. Catch basins that received a “fail” score had either late-instar larvae and or pupae present regardless of treatment status. Catch basins received applications of *L. sphaericus* (VectoLex® FG).
(DOCX)

S5 Table Adult female mosquito trap counts in 2019. Total and mean number of adult female mosquitoes captured from gravid and CDC-baited light traps within each of the four 2.59 – km^2^ study sites in 2019. Three of the four sites (Sites 1, 2, and 3) received catch basin treatments of *L. sphaericus* (VectoLex® FG). *All other mosquito species other than *Cx. pipiens* or *Cx. restuans*. ** Specimens too damaged to be differentiated between *Cx. pipiens* and *Cx. restuans*.
(DOCX)

S6 Table General additive model results of the integrated abundance. Results from the general additive model of the integrated (area under the curve) abundance of (A) adult gravid *Cx. pipiens* and *Cx. restuans* collected in gravid traps in the four study sites and of (B) adult female host-seeking *Cx. pipiens* and *Cx. restuans* collected in CDC-baited light traps located at four study sites in the greater metropolitan area of Milwaukee, WI in 2019. Three of the four sites received catch basin treatments of *L. sphaericus* (VectoLex® FG). The gravid integrated abundance was modeled as the response variable, treatment duration was used as the main effect, and date of first larvicide treatment with the number of functional trap events as an offset. The host-seeking integrated abundance was modeled as the response variable and treatment duration was used as the main effect.
(DOCX)

S7 Table General additive model results of the stability of the integrated abundance. Results from the general additive model of the stability of the (A) integrated adult gravid *Cx. pipiens* and *Cx. restuans* abundance collected from gravid traps and (B) integrated adult host-seeking *Cx. pipiens* and *Cx. restuans* abundance collected from CDC-baited light traps in the four study sites in the greater metropolitan area of Milwaukee, WI in 2019. Three of the four sites received catch basin treatments of *L. sphaericus* (VectoLex® FG). The gravid stability (standard deviation of the abundance) was modeled as the response variable, treatment duration was used as the main effect, and date of first larvicide treatment with the number of functional trap events as an offset. The host-seeking stability of the integrated abundance was modeled as the response variable and treatment duration was used as the main effect.
(DOCX)
