# Supplementary figures and images for: Catch basin larvicide treatments impact adult mosquito West Nile virus vector species in metropolitan Milwaukee, WI, U.S.A
Source: PLoS One. 2026 Apr 15;21(4):e0342150. doi: 10.1371/journal.pone.0342150 (PMC13082594; doi:10.1371/journal.pone.0342150)

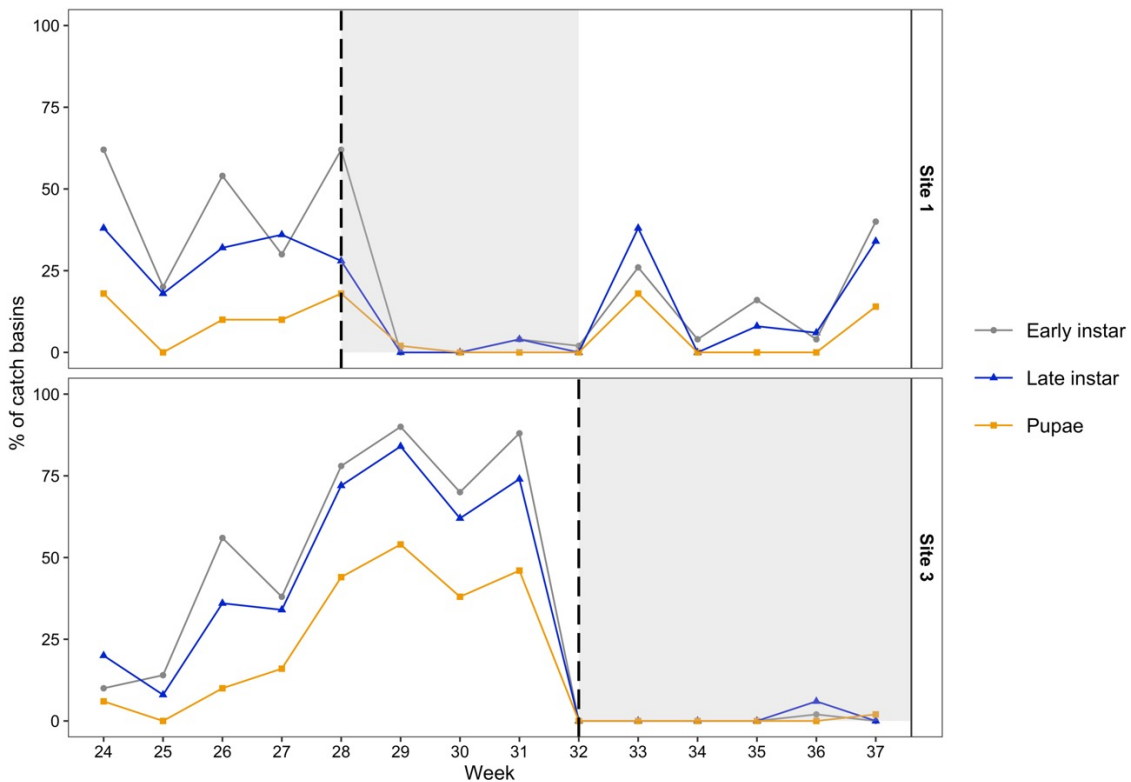

Supplement: S1 Fig — Weekly percent of catch basins inspected with early instar (first and second instar), late instar (third and fourth instar), and pupae in 2018. Catch basin larvicide applications with L. sphaericus illustrated by dashed vertical black lines and grey frames display when site was under treated conditions. (PDF) [file pone.0342150.s001.pdf]

A.

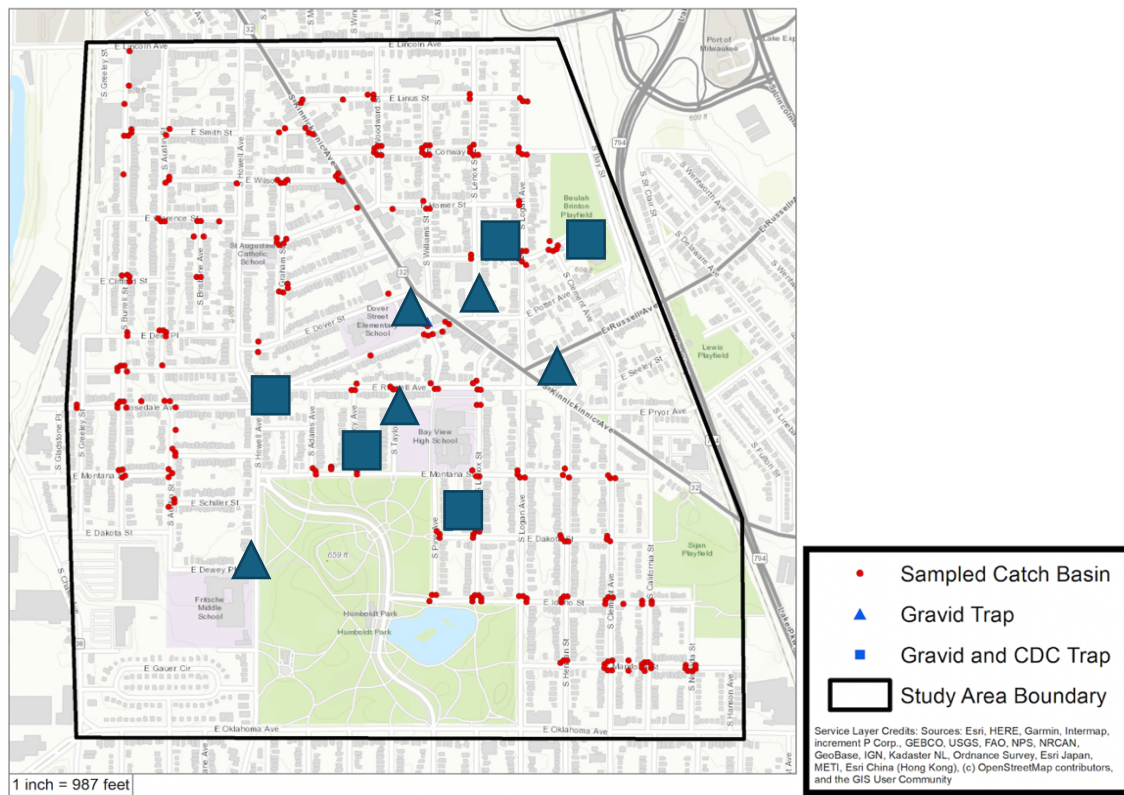

**B.**

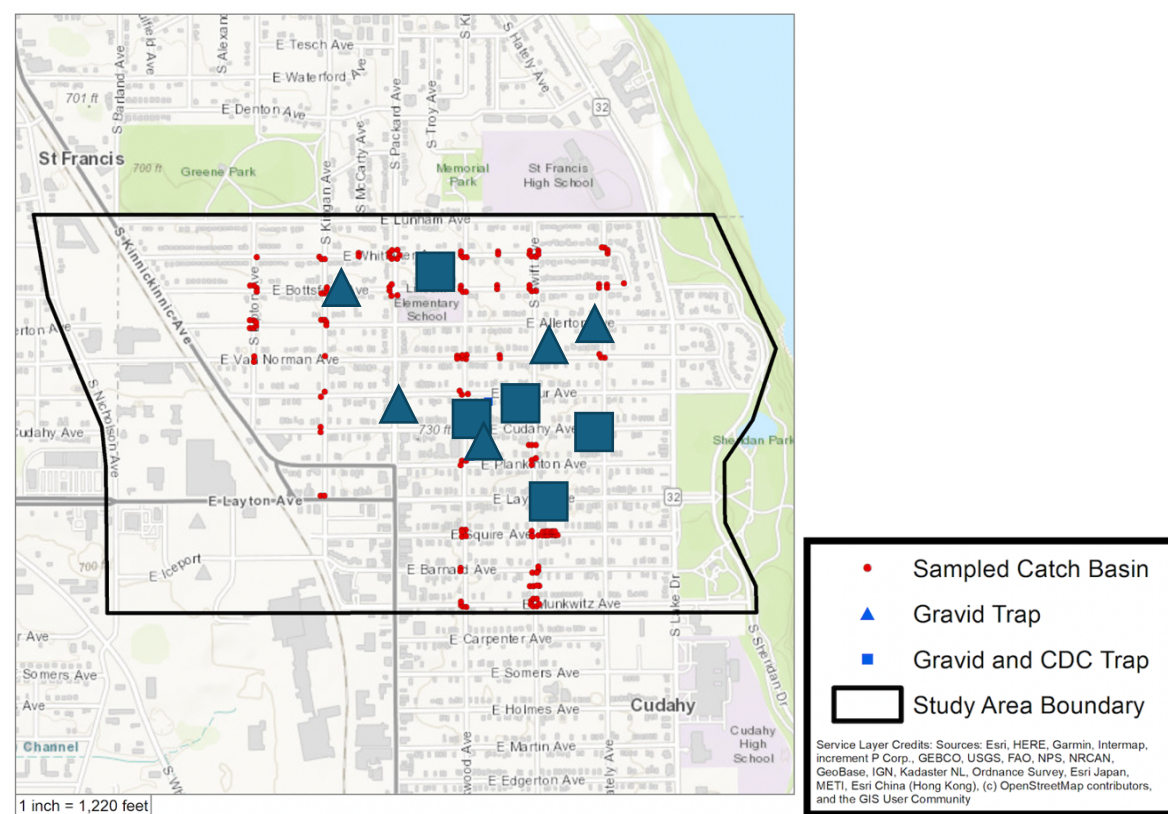

C.

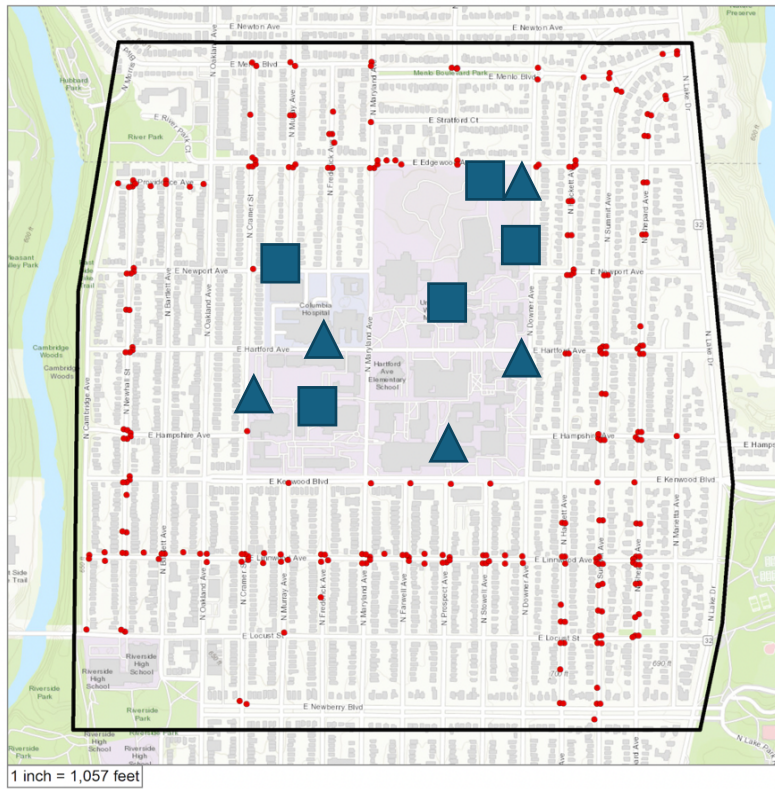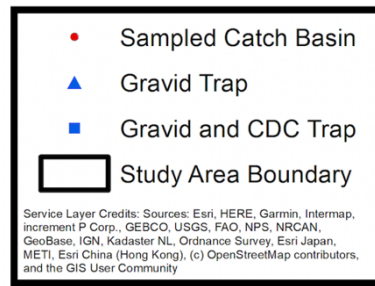

D.

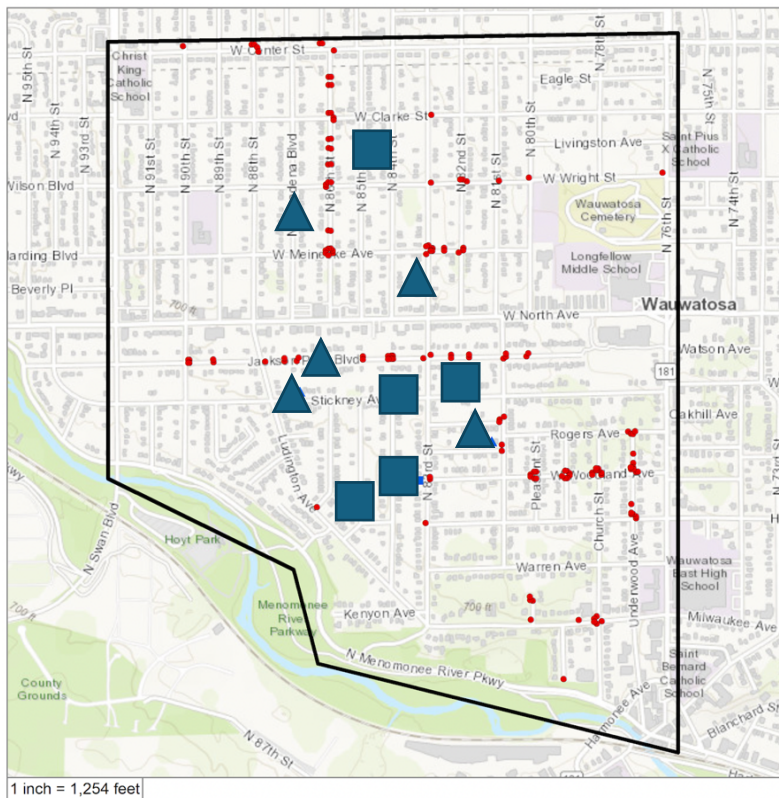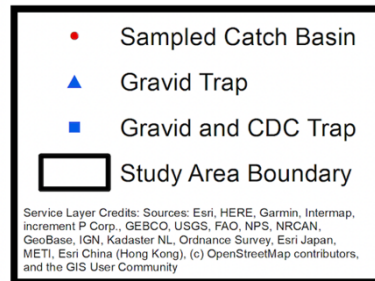

Supplement: S2 Fig — Adult mosquito gravid (blue triangle) and baited-CDC light trap (blue square) trapping locations. Inspected subset of catch basins (red circle) used in weekly evaluations; catch basins not visited in weekly evaluations not displayed in figure. (A) Site 1, Bay View, WI. A total of 952 catch basins were identified and treated within the 2.59 – km2 site boundary. (B) Site 2, Cudahy, WI. A total of 748 catch basins were identified and treated within the 2.59 – km2 site boundary. (C) Site 3, University of Wisconsin-Milwaukee campus, Milwaukee, WI. A total of 806 catch basins were identified and treated within the 2.59 – km2 site boundary. (D) Site 4, Wauwatosa, WI. A total of 782 catch basins were identified and treated within the 2.59 – km2 site boundary. (PDF) [file pone.0342150.s002.pdf]
