# Supplementary material for: Catch basin larvicide treatments impact adult mosquito West Nile virus vector species in metropolitan Milwaukee, WI, U.S.A
Source: PLoS One. 2026 Apr 15;21(4):e0342150. doi: 10.1371/journal.pone.0342150 (PMC13082594; doi:10.1371/journal.pone.0342150)
Supplement: S3 Table — (DOCX) [file pone.0342150.s003.docx]

|  | **Site 1** | | **Site 2** | | **Site 3** | | **Site 4** | | **Total** | |
| --- | --- | --- | --- | --- | --- | --- | --- | --- | --- | --- |
| Mosquito Species | No. larvae | % | No. larvae | % | No. larvae | % | No. larvae | % | No. larvae | % |
| *Cx. pipiens* | 35 | 7.8 | 18 | 4.7 | 154 | 24.0 | 662 | 44.9 | 869 | 29.5 |
| *Cx. restuans* | 408 | 91.5 | 364 | 95.3 | 484 | 75.6 | 811 | 55.1 | 2,067 | 70.3 |
| *Ae. vexans* | 3 | 0.7 | 0 | - | 1 | 0.2 | 0 | - | 4 | 0.1 |
| *Ae. japonicus* | 0 | - | 0 | - | 1 | 0.2 | 0 | - | 1 | 0.03 |
| *Cs. inornata* | 0 | - | 0 | - | 1 | 0.2 | 0 | - | 1 | 0.03 |
| **Total** | 446 |  | 382 |  | 641 |  | 1,473 |  | 2,942 |  |
