# Supplementary material for: Catch basin larvicide treatments impact adult mosquito West Nile virus vector species in metropolitan Milwaukee, WI, U.S.A
Source: PLoS One. 2026 Apr 15;21(4):e0342150. doi: 10.1371/journal.pone.0342150 (PMC13082594; doi:10.1371/journal.pone.0342150)
Supplement: S4 Table — Number of observed catch basins with early-instar larvae (first and second), late-instar larvae (third and fourth), pupae, and number of pupae observed during weekly inspections at each of the four 2.59 – km2 sites located in the greater Milwaukee, WI area in 2019. Catch basins that received a “fail” score had either late-instar larvae and or pupae present regardless of treatment status. Catch basins received applications of L. sphaericus (VectoLex® FG). (DOCX) [file pone.0342150.s004.docx]

**S4 Table**

| **Site** | **Inspection week** | **Treat status** | **No. of catch basins** | | | | | **Total pupae observed** |
| --- | --- | --- | --- | --- | --- | --- | --- | --- |
|  |  |  | early instar | late instar | pupae | "fail" score | total evaluated |  |
| Site 1 | 27 | not treated | 20 | 13 | 9 | 14 | 30 | 69 |
| Site 2 | 27 | not treated | 17 | 13 | 4 | 14 | 30 | 11 |
| Site 3 | 27 | not treated | 12 | 4 | 2 | 6 | 30 | 3 |
| Site 4 | 27 | not treated | 4 | 7 | 5 | 9 | 30 | 11 |
| Site 1 | 28 | not treated | 19 | 21 | 14 | 22 | 30 | 277 |
| Site 2 | 28 | not treated | 11 | 14 | 2 | 15 | 30 | 2 |
| Site 3 | 28 | not treated | 14 | 19 | 7 | 20 | 30 | 29 |
| Site 4 | 28 | not treated | 25 | 28 | 14 | 28 | 30 | 141 |
| Site 1 | 29 | treated | 0 | 0 | 0 | 0 | 30 | 0 |
| Site 2 | 29 | not treated | 20 | 23 | 10 | 23 | 30 | 66 |
| Site 3 | 29 | not treated | 14 | 9 | 6 | 9 | 30 | 22 |
| Site 4 | 29 | not treated | 16 | 20 | 11 | 20 | 30 | 76 |
| Site 1 | 30 | treated | 1 | 1 | 1 | 2 | 30 | 1 |
| Site 2 | 30 | not treated | 20 | 3 | 1 | 3 | 30 | 1 |
| Site 3 | 30 | not treated | 16 | 8 | 8 | 10 | 30 | 41 |
| Site 4 | 30 | not treated | 13 | 8 | 8 | 9 | 30 | 35 |
| Site 1 | 31 | treated | 7 | 7 | 7 | 7 | 30 | 75 |
| Site 2 | 31 | treated | 0 | 1 | 0 | 1 | 30 | 0 |
| Site 3 | 31 | not treated | 25 | 23 | 13 | 23 | 30 | 74 |
| Site 4 | 31 | not treated | 30 | 30 | 27 | 30 | 30 | 402 |
| Site 1 | 32 | treated | 0 | 0 | 0 | 0 | 30 | 0 |
| Site 2 | 32 | treated | 0 | 0 | 0 | 0 | 30 | 0 |
| Site 3 | 32 | not treated | 23 | 19 | 15 | 20 | 30 | 157 |
| Site 4 | 32 | not treated | 20 | 21 | 7 | 21 | 30 | 20 |
| Site 1 | 33 | treated | 19 | 15 | 3 | 16 | 30 | 178 |
| Site 2 | 33 | treated | 0 | 0 | 0 | 0 | 30 | 0 |
| Site 3 | 33 | treated | 2 | 3 | 2 | 3 | 30 | 4 |
| Site 4 | 33 | not treated | 30 | 30 | 28 | 30 | 30 | 1137 |
| Site 1 | 34 | treated | 0 | 0 | 1 | 1 | 30 | 1 |
| Site 2 | 34 | treated | 2 | 2 | 1 | 3 | 30 | 3 |
| Site 3 | 34 | treated | 2 | 2 | 3 | 3 | 30 | 4 |
| Site 4 | 34 | not treated | 29 | 29 | 23 | 29 | 30 | 249 |
| Site 1 | 35 | treated | 0 | 0 | 0 | 0 | 30 | 0 |
| Site 2 | 35 | treated | 0 | 0 | 0 | 0 | 30 | 0 |
| Site 3 | 35 | treated | 0 | 0 | 0 | 0 | 30 | 0 |
| Site 4 | 35 | not treated | 27 | 22 | 6 | 23 | 30 | 17 |
