# Supplementary material for: Catch basin larvicide treatments impact adult mosquito West Nile virus vector species in metropolitan Milwaukee, WI, U.S.A
Source: PLoS One. 2026 Apr 15;21(4):e0342150. doi: 10.1371/journal.pone.0342150 (PMC13082594; doi:10.1371/journal.pone.0342150)
Supplement: S5 Table — Total and mean number of adult female mosquitoes captured from gravid and CDC-baited light traps within each of the four 2.59 – km2 study sites in 2019. Three of the four sites (Sites 1, 2, and 3) received catch basin treatments of L. sphaericus (VectoLex® FG). *All other mosquito species other than Cx. pipiens or Cx. restuans. ** Specimens too damaged to be differentiated between Cx. pipiens and Cx. restuans. (DOCX) [file pone.0342150.s005.docx]

|  | | **Site 1** | | **Site 2** | | **Site 3** | | **Site 4** | |
| --- | --- | --- | --- | --- | --- | --- | --- | --- | --- |
|  |  | No. female  adults | Mean per trap  (n = 352) | No. adults | Mean per trap  (n = 349) | No. adults | Mean per trap  (n = 352) | No. adults | Mean per trap  (n = 347) |
| Gravid trap | Undifferentiated  *Cx. pipiens* and  *Cx. restuans* | 45,063 | 127.66 | 48,482 | 138.92 | 77,026 | 218.82 | 73,707 | 212.41 |
|  | Other * | 784 | 2.23 | 579 | 1.66 | 1,398 | 3.97 | 1,510 | 4.35 |
|  | **Total** | 45,848 | 130.25 | 49,061 | 140.58 | 78,424 | 222.80 | 75,217 | 216.76 |
|  | | | | | | | | | |
|  | | No.  Female  adults | Mean per trap  (n = 171) | No. adults | Mean per trap  (n = 172) | No. adults | Mean per trap  (n = 176) | No. adults | Mean per trap  (n = 175) |
| CDC-baited light trap | *Cx. pipiens* | 225 | 1.49 | 179 | 1.04 | 497 | 2.82 | 623 | 3.56 |
|  | *Cx. restuans* | 1,157 | 6.77 | 1,341 | 7.80 | 2,815 | 8.00 | 5,488 | 31.36 |
|  | *Cx. pipiens/restuans*** | 429 | 2.51 | 452 | 2.63 | 1,002 | 5.69 | 1,598 | 9.13 |
|  | Other * | 2,343 | 13.70 | 2,747 | 15.97 | 3,263 | 18.54 | 7,242 | 41.38 |
|  | **Total** | 4,358 | 25.49 | 4,992 | 29.02 | 8,082 | 45.92 | 15,926 | 91.00 |

**S5 Table**
