# Supplementary material for: Catch basin larvicide treatments impact adult mosquito West Nile virus vector species in metropolitan Milwaukee, WI, U.S.A
Source: PLoS One. 2026 Apr 15;21(4):e0342150. doi: 10.1371/journal.pone.0342150 (PMC13082594; doi:10.1371/journal.pone.0342150)
Supplement: S6 Table — Results from the general additive model of the integrated (area under the curve) abundance of (A) adult gravid Cx. pipiens and Cx. restuans collected in gravid traps in the four study sites and of (B) adult female host-seeking Cx. pipiens and Cx. restuans collected in CDC-baited light traps located at four study sites in the greater metropolitan area of Milwaukee, WI in 2019. Three of the four sites received catch basin treatments of L. sphaericus (VectoLex® FG). The gravid integrated abundance was modeled as the response variable, treatment duration was used as the main effect, and date of first larvicide treatment with the number of functional trap events as an offset. The host-seeking integrated abundance was modeled as the response variable and treatment duration was used as the main effect. (DOCX) [file pone.0342150.s006.docx]

**S6 Table**

**A.**

| **Variable** | **Est.** | **Std. Err.** | **z-value** | **Pr(> \|z\|)** |
| --- | --- | --- | --- | --- |
| Intercept | 2.66 | 3.10 | 0.86 | 0.3898 |
| TreatmentDurationDays | -0.10 | 0.05 | -2.06 | 0.0391 |
| WeekFirstTreatment | -0.86 | 0.11 | -7.60 | < 0.001 |

**B.**

| **Variable** | **Est.** | **Std. Err.** | **z-value** | **Pr(> \|z\|)** |
| --- | --- | --- | --- | --- |
| Intercept | -27.85 | 0.38 | -73.93 | < 0.001 |
| TreatmentDurationDays | -0.004 | 0.01 | -0.34 | 0.732 |
