# Supplementary material for: Catch basin larvicide treatments impact adult mosquito West Nile virus vector species in metropolitan Milwaukee, WI, U.S.A
Source: PLoS One. 2026 Apr 15;21(4):e0342150. doi: 10.1371/journal.pone.0342150 (PMC13082594; doi:10.1371/journal.pone.0342150)
Supplement: S7 Table — Results from the general additive model of the stability of the (A) integrated adult gravid Cx. pipiens and Cx. restuans abundance collected from gravid traps and (B) integrated adult host-seeking Cx. pipiens and Cx. restuans abundance collected from CDC-baited light traps in the four study sites in the greater metropolitan area of Milwaukee, WI in 2019. Three of the four sites received catch basin treatments of L. sphaericus (VectoLex® FG). The gravid stability (standard deviation of the abundance) was modeled as the response variable, treatment duration was used as the main effect, and date of first larvicide treatment with the number of functional trap events as an offset. The host-seeking stability of the integrated abundance was modeled as the response variable and treatment duration was used as the main effect. (DOCX) [file pone.0342150.s007.docx]

**S7 Table**

**A.**

| **Variable** | **Est.** | **Std. Err.** | **z-value** | **Pr(> \|z\|)** |
| --- | --- | --- | --- | --- |
| Intercept | -0.97 | 1.05 | -0.922 | 0.3565 |
| TreatmentDurationDays | -0.11 | 0.05 | -2.29 | 0.0218 |
| WeekFirstTreatment | -0.86 | 0.07 | -12.85 | < 0.001 |

**B.**

| **Variable** | **Est.** | **Std. Err.** | **z-value** | **Pr(> \|z\|)** |
| --- | --- | --- | --- | --- |
| Intercept | -31.92 | 0.35 | -91.20 | < 0.001 |
| TreatmentDurationDays | -0.01 | 0.01 | -0.73 | 0.466 |
